# Effect of Biologically Oriented Preparation Technique on the Stress Concentration of Endodontically Treated Upper Central Incisor Restored with Zirconia Crown: 3D-FEA

**DOI:** 10.3390/molecules26206113

**Published:** 2021-10-10

**Authors:** Luigi Giovanni Bernardo Sichi, Fernanda Zapater Pierre, Laura Viviana Calvache Arcila, Guilherme Schmitt de Andrade, João Paulo Mendes Tribst, Pietro Ausiello, Alessandro Espedito di Lauro, Alexandre Luiz Souto Borges

**Affiliations:** 1 Department of Dental Materials and Prosthodontics, Institute of Science and Technology, São Paulo State University (Unesp), São José dos Campos 12245-000, Brazil; luigi.sichi@gmail.com (L.G.B.S.); fernanda.pierre@unesp.br (F.Z.P.); laura.calvache@unesp.br (L.V.C.A.); alexanborges@gmail.com (A.L.S.B.); 2 Faculty of Dentistry, Universidade Estadual do Oeste do Paraná (UNIOESTE), Cascavel 85819-110, Brazil; guisdandrade@hotmail.com; 3 Department of Dentistry, University of Taubaté (UNITAU), Taubaté 12020-270, Brazil; joao.tribst@gmail.com; 4 School of Dentistry, University of Naples Federico II, via S. Pansini 5, 80131 Naples, Italy; alessandroespedito.dilauro@unina.it

**Keywords:** dental restoration failure, finite element analysis, dental materials

## Abstract

The aim of this study was to evaluate the effect of biologically oriented preparation technique on the stress concentration of endodontically treated upper central incisors restored with zirconia crown (yttria-stabilized zirconia polycrystalline ceramic) through finite element analysis (FEA). Four models of maxillary central incisors containing enamel, dentin, periodontal ligament, cortical and medullary bone were created in CAD. Each model received a polymeric core-build up with nanofilled dental resin composite. The evaluated models were SM—preparation in shoulder 90°; CM—chamfer preparation; BOPT—biologically oriented preparation technique and BOPTB—BOPT preparation 1 mm below the cement-enamel junction. All models received zirconia crowns (5Y-TZP), fiberglass post and 1 mm ferrule. The models were imported into the analysis software with parameters for mechanical structural testing using the maximum principal stress and the tensile strength as the analysis criteria. Then, load of 150 N was applied at the cingulum with 45° slope to the long axis of the tooth, with the fixed base for each model. The type of marginal preparation affected the stresses concentration in endodontically treated teeth and in the zirconia crown margin. Considering the stress magnitude only, BOPT is a viable option for anterior monolithic zirconia crowns; however, with the highest stress magnitude at the restoration margin.

## 1. Introduction

A standard protocol for endodontic treated teeth has not yet been evidenced in the current literature. Nevertheless, if the dentist considers each case’s individuality, evaluating the amount of dental remnant, the restorative materials options, and the supporting tissue condition, the dentist may indicate treatments supported by scientific evidence [1,2,3].

In situations where there is limited tooth remnant to support the core build-up, intraradicular retainers (IR) may be necessary to ensure retention and support for the final restoration [4,5].

Regarding the final restoration in endodontically treated teeth, the literature shows that crowns would bring greater longevity to the dental element [6]. The ferrule effect and the effective sealing of the root canal prevent microorganisms infiltration that could compromise the root filling [7].

For this, the restoration must have a thin and uniform internal cement layer and marginal misfit of up to 120 μm [8,9], to avoid infiltration by microorganisms and solubilization of cement by saliva, which would lead to irritation of periodontal tissues [10]. Thus, the type of marginal preparation is a crucial factor because it can directly influence the restoration settlement, marginal adaptation [9,10], and fracture resistance [11].

However, the literature is scarce when it correlates the type of marginal preparation with the biomechanical effects in teeth treated endodontically, and that required IRs; often focusing on preparations for metal crowns [12] or on the type of marginal preparation that would guarantee the best ferrule effect [7,13]. In addition, new proposals for marginal preparation have been emerging, such as the BOPT biologically oriented preparation technique), indicated mainly in cases where there is a need to replace old restorations [14,15,16], and, even if there are already case reports about it, more studies are needed to better understand its biomechanical behavior [14,15,16].

BOPT is a vertical preparation technique without a finishing line that has as main objective to bring to the rehabilitated teeth the emergence profile similar to the natural teeth [14,15,16]. Therefore eliminating finishing lines in previously restored teeth or correcting the cement-enamel junction (CEJ) in unprepared teeth, with the possibility of repositioning the prosthesis edges at different gingival levels of up to 1 mm. Despite being a more complex technique, which requires a certain learning curve, it has the advantages of greater preservation of the dental substrate, greater gingival stability, ease of molding, and increased prosthetic retention [15].

Therefore, the objective of this study was to evaluate the influence of the type of marginal preparation (BOPT, chamfer, and shoulder) through finite element analysis (FEA) on stress distribution in teeth treated endodontically, with fiberglass post and restored with full zirconia crowns. The null hypothesis of this study is that there is no difference between the types of preparation in the stress distribution.

## 2. Methods

**Figure 1:**
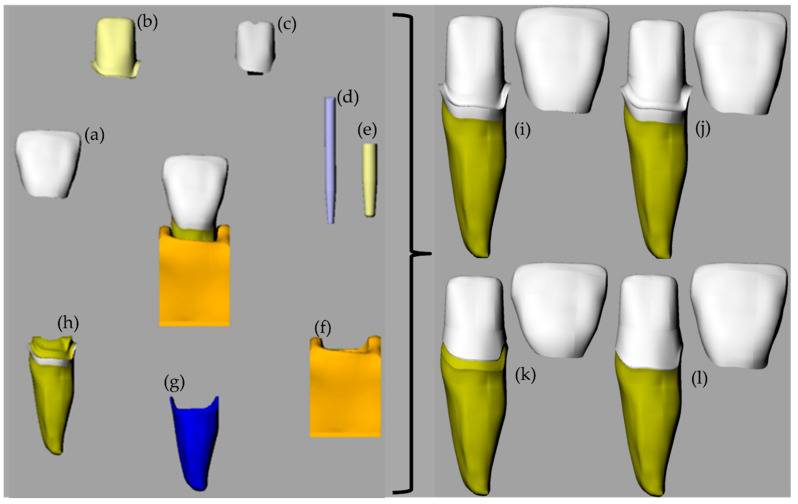
The geometric features of the analyzed model according to the preparation type. (**a**) Ceramic crown, (**b**) resin cement, (**c**) core build-up, (**d**) fiberglass post, (**e**) post cement, (**f**) bone tissue, (**g**) periodontal ligament, (**h**) root dentin and enamel, (**i**) SM model, (**j**) CM model, (**k**) BOPT model and (**l**) BOPTB model.

A 3D volumetric model of an upper central incisor was designed in CAD software (Rhinoceros version 5.0, McNeel North America, Seattle, WA, USA) following the BioCAD protocol [17,18,19] containing enamel, dentin, periodontal ligament of 0.2 mm, and cortical and medullary bone surrounding (Figure 1).

A perfect contact condition (shared nodes) was considered. The model was replicated four times to perform the modifications required for each preparation following the descriptions below:

- SM model—preparation in 90° shoulder in CEJ with marginal thickness of 0.5 mm and incisal reduction of 2 mm; zirconia crown with 1 mm thickness and cement layer in 0.1 mm; fiberglass post number 02 Exact (Angelus© Londrina, Brazil) with cement layer in 0.3 mm and remaining coronary (ferrule) with 1 mm height, 360° around the element.
- CM model—preparation with chamfer in CEJ with marginal thickness of 0.5 mm and incisal reduction of 2 mm; zirconia crown with 1 mm thickness and cement layer in 0.1 mm; fiberglass post number 02 Exact (Angelus© Londrina, Brazil) with cement layer in 0.3 mm and remaining coronary (ferrule) with 1 mm height, 360° around the element.
- BOPT model—preparation BOPT in CEJ with marginal thickness of 0.5 mm and incisal reduction of 2 mm; zirconia crown with 1 mm thickness and cement layer in 0.1 mm; fiberglass post number 02 Exact (Angelus© Londrina, Brazil) with cement layer in 0.3 mm and remaining coronary (ferrule) with 1 mm height, 360° around the element.
- BOPTB model—preparation similar to BOPT, positioned 1 mm below the CEJ with marginal thickness of 0.5 mm and incisal reduction of 2 mm; zirconia crown with 1 mm thickness and cement layer in 0.1 mm; fiberglass post number 02 Exact (Angelus© Londrina, Brazil) with cement layer in 0.3 mm and remaining coronary (ferrule) with 1 mm height, 360° around the element.
  BOPTB model—preparation similar to BOPT, positioned 1 mm below the CEJ with marginal thickness of 0.5 mm and incisal reduction of 2 mm; zirconia crown with 1 mm thickness and cement layer in 0.1 mm; fiberglass post number 02 Exact (Angelus© Londrina, Brazil) with cement layer in 0.3 mm and remaining coronary (ferrule) with 1 mm height, 360° around the element.

**Figure 2:**
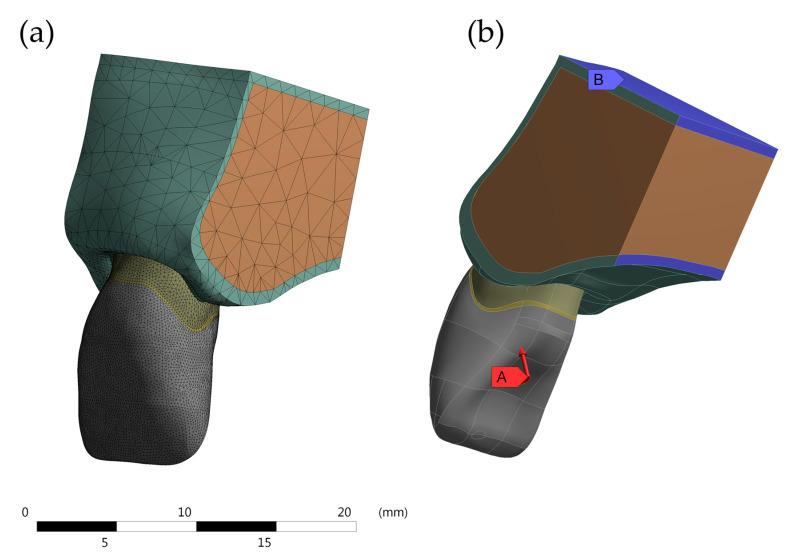
(**a**) Mesh division during the numerical approach, (**b**) boundary conditions applied in the present simulation (A—Loading and B—Fixation).

**Figure 3:**
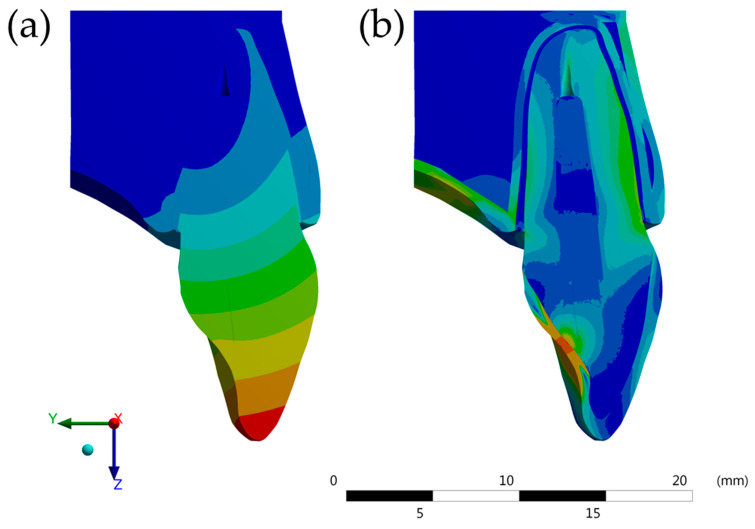
Meshing convergence representative results. (**a**) Total deformation and (**b**) von-Mises stress.

The models were imported in STEP format for the analysis software (ANSYS 17.2, ANSYS Inc., Houston, TX, USA) and passed the mesh convergence test, composed of 1,299,634 nodes and 833,462 tetragonal elements in average (Figure 2). The convergence test was performed according to the total deformation and Von Mises’ stress field (Figure 3).

The tests were based on parameters for mechanical structural test and maximum principal stress, and tensile was the failure criteria. The materials in ANSYS were considered isotropic, elastic, and homogeneous. The connections between cement and other surfaces were considered linear [20]. The mechanical properties are summarized in Table 1.

For each model, a load of 150 N was applied in the cingulum with an inclination of 45° in relation to the long axis of the tooth with bone base fixation. The results were evaluated for stress distribution based on the maximum principal stress on the root, post, cement and crown. Colorimetric stress maps were used to represent the stress field in the structures and to report each component’s behavior qualitatively.

## 3. Results

The coherence results show that there was no contact failure in the models tested, neither between the surfaces of the structures nor in the distribution of force applied (Figure 3).

**Figure 4:**
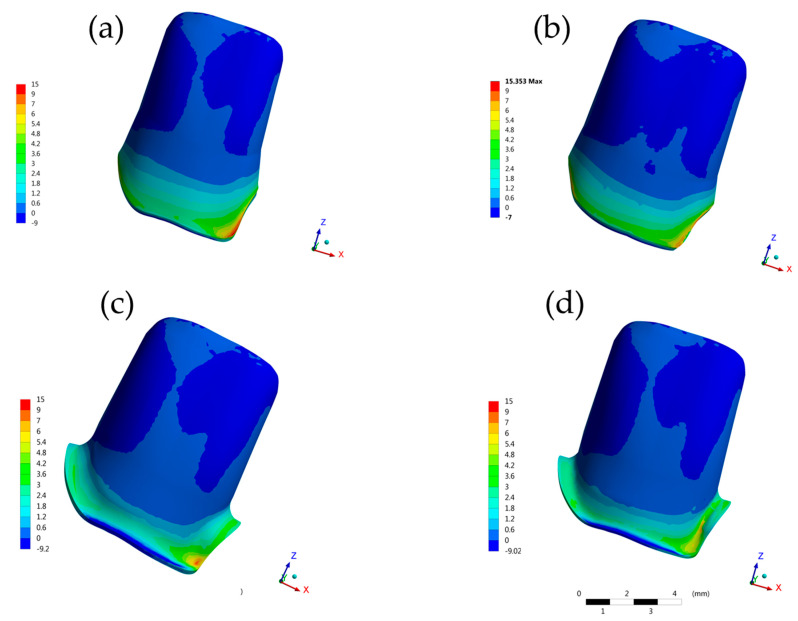
Buccal view of contour plots of each restorative procedure First principal stress (MPa) in the crown cement layer. (**a**) BOPTB, (**b**) BOPT, (**c**) CM and (**d**) SM.

**Figure 5:**
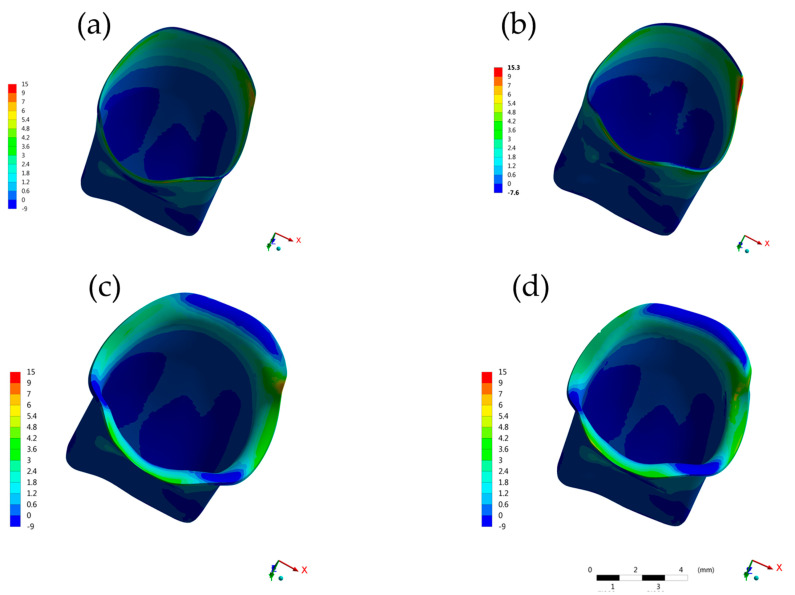
Bottom view of contour plots of each restorative procedure First principal stress (MPa) in the crown cement layer. (**a**) BOPTB, (**b**) BOPT, (**c**) CM and (**d**) SM.

**Figure 6:**
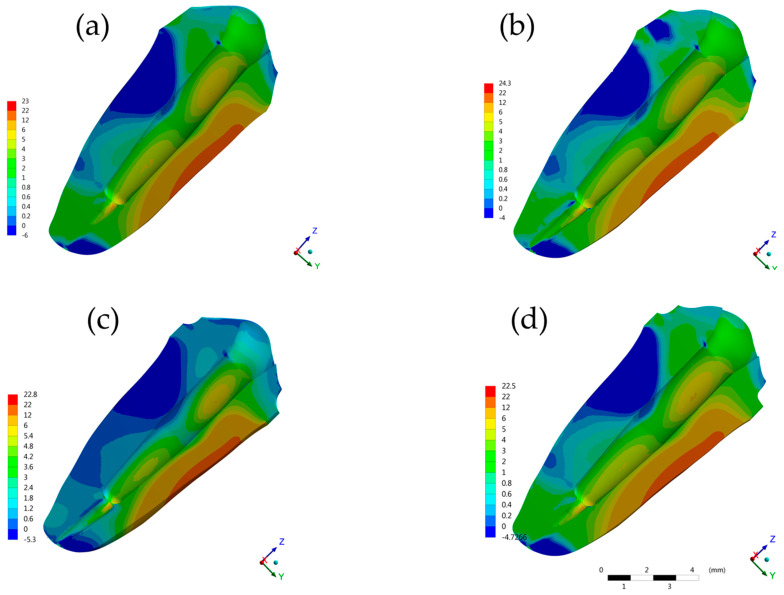
Contour plots of each restorative procedure first principal stress (MPa) in the root dentin. (**a**) BOPTB, (**b**) BOPT, (**c**) CM and (**d**) SM.

**Figure 7:**
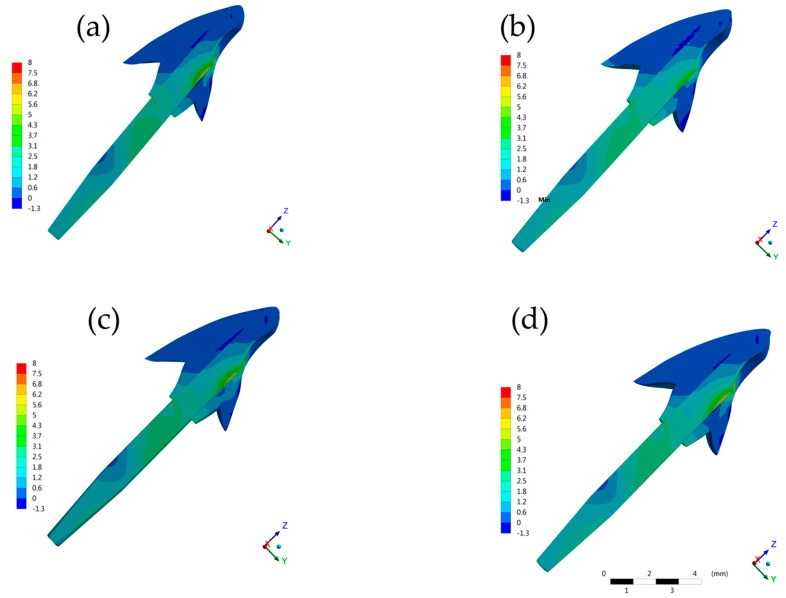
Contour plots of each restorative procedure first principal stress (MPa) in the post and core setup. (**a**) BOPTB, (**b**) BOPT, (**c**) CM and (**d**) SM.

The maximum principal stress analyzed in the cement (Figure 4 and Figure 5), on the root dentin (Figure 6), and in the post system (Figure 7), showed that there was no discrepancy difference between the models, however, the BOPT model presented the highest stress peaks (Table 2). The models with the lowest values were SM followed by CM.

In the internal crown termination line, a higher stress concentration was observed in the BOPTB (59.04 MPa) followed by the BOPT (51.71 MPa) (Table 2).

In the Figure 6, it is possible to observe the stress distribution in a transverse plane view of the dentin root, at the adhesive dentin interface, where the BOPT model obtained a higher stress magnitude (12.21 MPa) and the BOPTB model presented lower stress level (4.38 MPa). The SM group generally had the best performance, presenting the lowest values in the analysis performed, except at the adhesive dentin interface, followed by the CM group.

## 4. Discussion

The marginal preparation types can be divided into two distinct types, the horizontal (shoulder and chamfer) and the vertical (knife-edge and the shoulder variations and chamfer with bevel). Therefore the BOPT design should be defined as a vertical preparation, but that unlike conventional preparations with the marginal finish well defined by the dentist. In addition, the BOPT presents a “finish area”, and the dental technician modulates the new emergence profile to the restored tooth stipulating the restoration margin [21].

BOPT proposal is for eliminating CEJ in unprepared teeth or change the finish line of prepared teeth to create a new emergence profile proper for a more aesthetic gingival configuration for the patient, requiring a simultaneous study on gingiva and tooth during the execution protocol [14,15,16].

The literature does not define BOPT as a knife-edge preparation [14]; however, we believe it would be necessary to bind it to a knife-edge design due to the minimal thickness configuration required for the ceramic restoration. This study used a zirconia restauration, where categorically uses BOPT as a preparation option, demonstrating that the effect generated in the restorative material can be negative depending on the ceramic used.

To determine clinical acceptability in indirect restorations, parameters such as internal cement layer thickness [20,22] and marginal adaptation are some of the most used factors, due to their influence in the restoration fracture resistance and periodontal responses, which generally dictate the prosthesis survival [23]. Regarding the root dentin fractures [24], the present study did not show a difference between the simulated models suggesting that the crown preparation would not affect this region of the remaining tooth.

A study that compared knife-edge configuration to shoulder and chamfer preparation in zirconia crowns, showed better marginal adaptation with knife-edge configuration [25]. To show the marginal adaptation with other restorative materials, Gavelis et al., [9] showed that the type of marginal preparation, statistically influenced the marginal misfit values of metal crowns, and the preparations of the knife-edge type or parallel bevel in preparations type shoulder or chamfer presented smaller marginal spaces when compared to preparations on 90° shoulder and its bevel variations at 45° and 35°. However, the authors observed that the preparations on 90° shoulder had a better restoration settlement, with lower thickness in the occlusal cement layer, when compared to those of the knife-edge type.

For ceramic crowns in lithium disilicate, the knife-edge configuration presented the worst result of marginal adaptation and the best result in internal adaptation when comparing the effects of three different types of preparation: knife-edge, shoulder and chamfer [26].

The differences between knife-edge preparation compared to shoulder or chamfer preparations seem logical, since its configurations are visibly and theoretically distinguishable, mainly as to its indication and level of dental wear, however due to the lack of consensus in the literature, the definition of what is a shoulder preparation and a chamfer preparation is not always clear [27].

The chamfer preparation is classically described as a 90° concavity with rounded internal angle made by a diamond bur, on the other hand, the best-known variation of shoulder, 90° shoulder, as implied in its name, would have the internal angle of 90° in its concavity [28].

It is not clear in the literature yet whether shoulder or chamfer preparation would have better marginal and internal misfit results. Some studies point to the shoulder preparation with better results of marginal misfit, but chamfer preparation with better internal values [22], also for the shoulder preparation were found better marginal results for zirconia crowns [29]. Previous authors [30] did not found statistical difference between the two types of preparations. Even though the present study is not influenced by human interferences in marginal and internal misfits, as they are delimited and maintained in the CAD design and analysis software, it is important to emphasize that the cement layer influences the fracture resistance of fixed partial prostheses [31], as well as the bond strength of cement, can be influenced by the cement thickness [32]. These factors are corroborated by several studies in FEA that indicate that the failure risks are not precisely in the crown, but in the weakest part of the assembly (sometimes the cement) and the higher modulus of elasticity of the crown restorative material, would be better to protect the cement layer [33,34,35]. The use of zirconia crowns for BOPT and other types of preparation, as demonstrated in the results, can be seen as a positive factor for survival.

The present study showed a subtle difference between the four preparations designs regardless the analyzed region. Therefore its influence in the clinical behavior should not be significant. These results may be associated with the study of Kasem et al. [36], did not find a statistical difference between horizontal and vertical preparations in fracture resistance, regardless of the material, but zirconia crowns were the ones with higher fracture resistance and with high chances of catastrophic fractures. This dualist behavior occurs because the brittle behavior of this structured polycrystalline ceramic, that present toughening mechanism during fracture mechanics and will fail in many fragments. However, some studies point to a statistically significant difference between chamfer and knife-edge for zirconia crowns, being the knife-edge the configuration with the best result in fracture resistance [37]. For Beuer et al. [38] when considering zirconia crowns, shoulder and knife-edge preparations should be used to improve the mechanical results, followed by the chamfer type.

The similarity between the results in the present study may also be linked to the maintenance of the remaining tooth with ferrule effect, as it is widely studied in the literature as an essential factor in the mechanical behavior of teeth that required IRs [39,40,41]. The slightly better result for the BOPT preparation 1 mm below the CEJ, observed in the marginal region, is in accordance with results that point to a better performance for higher ferrule walls [42] and conventional preparations with axial bevel associated with dental remnant for ferrule effect [43].

The finite element analysis (FEA) makes it possible to predict the mechanical and structural behavior of materials and biological structures through a non-destructive and mathematical approach [44]. In addition important oral conditions such as the microbiological aspects from each patient should be considered in further in vivo evaluations [45,46,47]. It is important to note that the present method considers the structures and materials as homogeneous, isotropic and with linear behavior, it may not be able to predict clinical situations such as the presence of defects in materials and interfaces. In addition, the FEA applied standard features during model development and simulation process [48], compiling parameters that may be important for the models reproducibility based in clinical descriptions, however the models were used only with indirect validation (based on data from clinical literature) and should be carefully interpreted [48]. Therefore, more in vitro investigations and randomized clinical trials may be needed to better predict the clinical behavior of the evaluated groups.

During the tooth preparations different shapes and designs can be achieved, therefore generate different results that no single mechanical test can truly reproduce what the relative to tooth loading encountered in the oral environment [49]. Therefore, it is important to address the survivability of the crown–tooth [49] as well as the stress field distribution and magnitude complex rather than an individual entity.

There are some limitations presents during this finite element analysis, such as the absence of nonlinear simulation of contacts and materials with viscoelastic behaviors [49]. In addition, due to its complexity of its structure, the mechanical properties of PDL are considered simplified. Importantly, the viscoelastic behavior, cyclic loading and fatigue have not been performed, which should be done in further studies [49].

## 5. Conclusions

It can be concluded that the type of marginal preparation affected the stresses concentration in endodontically treated teeth and in the zirconia crown margin. Considering the stress magnitude only, BOPT is a viable option for anterior monolithic zirconia crowns; however, with the highest stress magnitude at the restoration margin.

## Figures and Tables

**Table 1:** Material’s mechanical properties: Young’s modulus and Poisson ratio. Glass fiber post was simulated with orthotropic behavior.

Material	Elastic Modulus (GPa)		Poisson’s Ratio
Dentin	24.35		0.30
Enamel	84.82		0.30
Cortical bone	13.7		0.30
Medullary bone	1.37		0.30
Periodontal ligament	0.05		0.45
Resin core build-up	12.3		0.24
Resin cement	7		0.28
Zirconia	210		0.30
Glass fiber post	E	G	
	X = 37	XY = 3.1	XY = 0.27
	Y = 9.5	XZ = 3.5	XZ = 0.34
	Z = 9.5	YZ = 3.1	YZ = 0.27

**Table 2:** Stress peaks (MPa) in the crown margin and cervical dentin according to the preparation model.

Model	Region	Stress Peak
BOPT	Crown margin	51.71
	Cervical dentin	4.38
BOPTB	Crown margin	59.04
	Cervical dentin	12.21
CM	Crown margin	43.42
	Cervical dentin	7.65
SM	Crown margin	40.18
	Cervical dentin	6.01

## Data Availability

Data available on request to the corresponding author.
